# Antimicrobial Drug Resistance in *Salmonella enteritidis* Isolated From Edible Snakes With Pneumonia and Its Pathogenicity in Chickens

**DOI:** 10.3389/fvets.2020.00463

**Published:** 2020-08-04

**Authors:** Ying Xia, Hao Li, Yaoqin Shen

**Affiliations:** College of Veterinary Medicine, Huazhong Agricultural University, Wuhan, China

**Keywords:** *Salmonella enteritidis*, antimicrobial resistance, edible snakes, foodborne pathogen, pathogenicity

## Abstract

The growing consumption of snakes in China has led to a boom in edible snakes farming. Food producing reptiles, such as snakes can carry many pathogenic microbes and potentially infect humans. Here, we report the occurrence of multi drug resistant *Salmonella enteritidis* strains isolated from edible snakes in China. Our results showed that the isolated *S. enteritidis* was resistant to the majority of the tested drugs and sensitive to tetracycline and amikacin. Antimicrobial susceptibility test showed that the strains carried the blaTEM, qnrD, aadA_1_, catA_1_ o, sul I, and sul II genes. The pathogenicity testing of the *S. enteritidis* isolated strains showed that these strains were highly pathogenic (75% mortality, with LD_50_ at 10^7.7^ CFU/mL). The chickens in the high-dose groups developed acute septicemia and died within 24 h. Results of the dissection showed extensive abdominal bleeding and swelling in the high dose groups, as well as hyperemia edema in the livers, lungs, kidneys, cecum, and bursa of the chickens, with spotty bleeding. In addition, rod-shaped bacterial aggregation was also seen in the visual field. A total of 23 virulence genes, mainly associated with pathogenicity island were tested, of which 8 genes including avrA, iacP, prgK, ssrA, siiD (spi4D), siiE, spi4H, and pipC were found positive. Altogether, our results provide useful information regarding edible snakes contaminated with *S. enteritidis*, which may have public health implications.

## Introduction

*Salmonella* is one of the most common Enterobacteriaceae. Since its discovery in 1885, more than 2,600 *Salmonella* serotypes have been reported (1), making it one of the most common zoonotic pathogens in the world. *Salmonella* is the leading cause of morbidity and mortality in children under 5 years of age in most developing countries (2–4). In China, 22.2% of food poisoning was caused by *Salmonella*, and the vast majority was caused by eating meat products (5). The strains that caused 99% human and animal infections belong to *Salmonella enterica* ssp. *Enterica*, of which *S. typhimurium* and *S. enteritidis* are the most common serotypes (6). And they are also important foodborne pathogens (7). One of the main characteristics of *Salmonella enterica* is that it can cause a variety of diseases of varying degrees in different hosts, such as human, swine, cows, birds and mice. In humans, *S. enteritidis* can cause a variety of foodborne diseases such as gastroenteritis and systemic or persistent diseases (8–10).

Poultry and poultry products are considered as the main reservoir of *S. enteritidis* (11), but it is worth noting that reptiles are also an important host of *Salmonella*. In the United States, about 6% of cases of human *Salmonella* infection were from reptiles. In southwest England, contacting with reptiles has been linked to 27.4% of *Salmonella* cases in children under 5 years of age (12). According to published reports, the majority of human cases of reptile-related salmonellosis come from non-toxic reptiles kept as pets (13). In China, snake meat has a huge number of consumers because of its high nutrition and good taste. *Salmonella* carried by snakes can be transmitted to humans through the food chain, causing harm to humans. Studies have found that vipers may be reservoirs of *Salmonella* serotypes associated with human salmonellosis (14). European Union has planned to establish food safety standards related to *Salmonella* contamination in reptile meat. The European Commission is proposing to limit *Salmonella* in reptile meat, which is another reminder that the presence of *Salmonella* in reptile meat could pose a potential threat to humans.

*Salmonella enteritidis* is causing serious complications in human since 1980. In 2011, the total number of cases reported to CED increased significantly and *S. enteritidis* became more common in South Africa (15, 16). Despite global efforts to constrain the spread of *Salmonella, S. enteritidis* infections continue to spread rapidly, which brings challenges to global health systems. In many developed countries, the development of resistance to commonly used antibiotics against zoonotic pathogens is almost the inevitable consequence of the abused use of antibiotics in food-producing animals. We have widely agreed that zoonotic strains acquired resistance from food animals first then infected human being through food chains. *Salmonella* from clinical practice and rapid spread of multi-drug resistance on a global scale have attracted the attention worldwide. Despite the development of national antimicrobial susceptibility testing programs, bacterial resistance remains a problem that needs to be addressed as soon as possible, given the severity of foodborne *Salmonella* resistance.

At present, there are only few reports about wild snakes or pet snakes carrying pathogenic bacteria. Limited reports about pathogens from food source snakes have been discussed. This study is the first report on *S. enteritidis* isolates from snake suffering from pneumonia. The aim of this study was to isolate and evaluate the pathogenicity of the *S. enteritidis* strains and determine the fundamental information for public health safety and further studies.

## Materials and Methods

### Isolation and Purification of Pathogenic Bacteria

Under sterile conditions, the lungs of the snakes with pneumonia were collected. The internal structures of intestinal tissues were obtained with a sterile sterilizing ring, and the pathogens were separated by streaks on MAC agar (MacConkey). The culture medium was placed in a constant temperature incubator at 37°C for 18 h then the colony morphology was observed. Suspected bacterial colonies were selected by a sterile sterilizing ring and separated by streaking on a new MAC agar to be purified.

### Identification of Pathogenic Bacteria

The pathogens were identified by Gram staining, biochemical examination, and PCR (Polymerase Chain Reaction) analysis. A pair of specific primers were designed for the specific gene sequence of invA, the specific gene of *Salmonella*, and the expected amplified target fragment size was 374 bp. The invA gene PCR primer sequence was: invA-F 5′-GCTCTTTCGTCTGGCATTA-3′. invA R-5′-CGGCATAGCGTCACCTT-3′. PCR reaction conditions were pre-denaturation at 95°C for 5 min, denaturation at 95°C for 30 s. Annealed at 50°C and extended at 72°C for 1 min. There were 35 cycles of reaction and extended at 72°C for 10 min. The 7 μL PCR amplification product was subjected to 1% agarose gel electrophoresis, and the standard strain of *S. enteritidis* (NCTC13349) was used as the positive control. The strip was observed and preserved using a gel imaging system. Finally, PCR products were sent to Wuhan Jinkairui biological engineering co., LTD for sequencing.

### Identification of Serotypes

The serotype of *Salmonella* was identified by slide agglutination tests. According to the Kauffmann-White scheme (17), *Salmonella* diagnostic serum kit (MicroFast; LR70602) was used for testing.

### Antimicrobial Susceptibility Test

Disc diffusion test (K-B method) was used to conduct sensitivity tests for 19 antimicrobial drugs on isolated strains, and the whole process was developed, according to the standards of the American Association for Clinical and Laboratory Standards Institute (CLSI). Before antimicrobial resistant test, the purified strain was inoculated in TSB (tryptic soy broth) liquid culture for 18 h. One hundred micro-liters of fresh bacterial liquid were taken and placed on TSA (Tryptic Soy Agar) medium. The liquid was spread evenly with sterile cotton swabs. Each drug sensitive paper was clipped and attached to the medium with sterile tweezers by triplicate. All plates were placed upside down in an incubator at 37°C for 18–24 h. After the cultivation, the formation of bacteria-resistant zones was observed, the diameter of bacteria-resistant zone was measured, the average values were calculated, and judged as susceptible, intermediate or resistant (18, 19). The types and contents of selected antibacterial drugs are shown in Table 1.

**Table 1:** Antimicrobial sensitive paper name and inhibitory.

**Susceptibility paper**	**Content each**	**Susceptibility paper**	**Content each**
Florfenicol	30 μg	Cephradine	30 μg
Clindamycin	2 μg	Furazolidone	300 μg
Polymyxin B	300 IU	Lincomycin	2 μg
Cephalothin	30 μg	Kitasamycin	15 μg
Ofloxacin	5 μg	Erythromycin	15 μg
Streptomycin	10 μg	Norfloxacin	5 μg
Gentamicin	10 μg	Amoxicillin	20 μg
Doxycycline	30 μg	Kanamycin	30 μg
Ciprofloxacin	5 μg	Neomycin	30 μg
Ceftriaxone sodium	30 μg		

### Detection of Antimicrobial Resistance Genes

Relevant gene sequences published on NCBI and literature were searched (20, 21), then primers were designed. They were synthesized by Wuhan Jinkairui biological engineering co. LTD. The categories, names, and primer sequences of resistant genes are shown in Table 2. The capacity of each PCR reaction system was 20 μL. It contains PCR Mix 10 μL. The upstream and downstream primers were 1 μL each. While DNA template and dd water was 2 and 6 μL, respectively. The reaction parameters of all genes amplified by PCR instruments were: pre-denaturation 95°C for 5 min, denaturation 95°C for 1 min, annealing at 54°C for 30 s, extension at 72°C for 1 min, a total of 35 cycles, extension at 72°C for 10 min. The amplified products were analyzed by 1% agarose gel electrophoresis and photographed by a gel imaging system. Samples with positive PCR results were selected for sequencing. The sequencing results were compared with the existing sequences in Blast software in GenBank.

**Table 2:** Primer sequence for PCR of resistance genes.

**Categories**	**Resistance genes**	**Primer sequences**	**Size (bp)**
β-lactams	blaTEM	F:5′-ATGAGTATTCAACATTTTCG-3′	643
		R:5′-TTACCAATGCTTAATCAGTG-3′	
	blaSHV	F:5′-ATGCGTTATATTCGCCTGTG-3′	860
		R:5′-TTAGCGTTGCCAGTGCTCGA-3′	
	blaPSE	F:5′-ATGCTTTTATATAAAATGTG-3′	571
		R:5′-TCAGCGCGACTGTGATGTAT-3′	
	blaCTM-M	F:5′-ACGTTGCATATATCGACGTTG-3′	544
		R:5′-GCAGCGACTGTCGTACCCTAT-3′	
	blaOXA	F:5′-TCAACTTTCAAGATCGCA-3′	591
		R:5′-GTGTGTTAGAATGGTGA-3′	
4-quinolones	qnrA	F:5′-ATTTCTCACGCCAGGATTTG-3′	519
		R:5′-GATCGGCAAAGGTCAGGTCA-3′	
	qnrB	F:5′-GATCGTGAAAGCCAGAAAGG-3′	469
		R:5′-ACGATGCCTGGTAGTTGTCC-3′	
	qnrC	F:5′-ATTTCTCACAGGCAAACT-3′	666
		R:5′-CTGGAATAACAATCACCC-3′	
	qnrD	F:5′-TTTTCGCTAACTAACTCGC-3′	984
		R:5′-GAAAGGATAAACAGGCAAAT-3′	
	qnrS	F:5′-ACGACATTCGTCAACTGCAA-3′	417
		R:5′-TAAATTGGCACCCTGTAGGC-3′	
Aminoglycosides	aadA_1_	F:5′-TCATTCCGTGGCGTTATCC-3′	343
		R:5′-TCGGCAGCGACATCCTT-3′	
	aadA_2_	F:5′-TCATTCCGTGGCGTTATC-3′	369
		R:5′-GGGCAGGTAGGCGTTTTA-3′	
Tetracyclines	tetA	F:5′-TTTCGGGTTCGGGATGGT-3′	656
		R:5′-TCGCCGTGAAGAGGAG-3′	
	tetB	F:5′-TCATTGCCGATACCACCTC-3′	247
		R:5′-GATTGCGTCTCAACCCCTAC-3′	
Chloramphenicols	catA_1_	F:5′GCAAGATGTGGCGTGTTACGGTGAA-3′	258
		R:5′-TCATTAAGCATTCTGCCGACATGGA-3′	
Sulfonamides	*sul*	F:5′-TTCGGCATTCTGAATCTCAC-3′	822
		R:5′-ATGATCTAACCCTCGGTCTC-3′	
	*sul*	F:5′-CGGCATCGTCAACATAACC-3′	722
		R:5′-GTGTGCGGATGAAGTCAG-3′	

### Detection of Virulence Genes

The virulence gene sequence recorded on GenBank and related literature were searched (22–24), and 23 pairs of specific virulence gene primers were designed by Oligo6 software, as shown in Table 3. The primers were synthesized by Wuhan Jinkairui biological engineering co. LTD. Plasmid virulence genes: spvA and spvD. Virulence genes of SPI-1: invH, sipA, sopA, sopD, avrA, iacP, and prgK. Virulence genes of SPI-2: ssaB, ssaQ, sifA, sseL, ssrA, and ttrB. Virulence genes of SPI-3: misL, rmbA, rhuM, and sugR. Virulence genes of SPI-4: siiD (spi4D), siiE, and spi4H. Virulence genes of SPI-5: pipC. The volume of amplified virulence gene PCR system was 20 μL, PCR Mix 10 μL, upstream primer 1 μL, downstream primer 1 μL, DNA template 2 μL, and ddH_2_O 6 μL. PCR amplification procedures: pre-denaturation at 95°C for 5 min, denaturation at 94°C for 30–45 s, annealing at 52–58°C for 30 s, extension at 72°C for 1 min, reaction 35 cycles. Extend at 72°C for 10 min. The amplified products were analyzed by 1% agarose gel electrophoresis and photographed by a gel imaging system. Samples with positive PCR results were selected for sequencing. The sequencing results were compared with the existing sequences in Blast software in Genbank.

**Table 3:** Primers sequences for PCR of virulence genes.

**Categories**	**Virulence genes**	**Primer sequences**	**Size (bp)**
Plasmid virulence	spvA	F:5′-GCTAACTGTCGGGCAAAG-3′	432
		R:5′-GGACAATGGCACGAACCT-3′	
	spvD	F:5′- CCCCTGATGATGAGAAGT-3′	316
		R:5′-ACAGTGGGATTAGACAGC-3′	
SPI-1	InvH	F:5′-AGCAACTGGCCAACGCAAAT-3′	153
		R:5′-TGCAGTCTTTCATGGGCAGCAA-3′	
	sipA	F:5′- TTCCCCTTTTAGCCT-3′	243
		R:5′-ACCTCCACACCGTTC-3′	
	sopA	F:5′-ACCTGCCGACTGGGCTAAG-3′	347
		R:5′-ACGAGGGCTGTTGTTGTGT-3′	
	sopD	F:5′- ACGACCATTTGCGGCG-3′	1,291
		R:5′-GAGACACGCTTCTTCG-3′	
	avrA	F:5′-AATGGAAGGCGTTGAATCTG-3′	170
		R:5′-GAGCTGCTTTGGTCCTCAAC-3′	
	iacP	F:5′-CACCTCTTGTATTGCCGTTG-3′	176
		R:5′-GGCATATATCCGCAAAGGTC-3′	
	prgK	F:5′-TTGAACAGCGACTGGAACAG-3′	217
		R:5′-TCATAATCCACATCGGCAAA-3′	
SPI-2	ssaB	F:5′-ATGTCTGAGGAGGGAT-3′	382
		R:5′-GTTTATGGTGATTGCG-3′	
	ssaQ	F:5′-GAATAGCGAATGAAGAGCGTCGTCC-3′	455
		R:5′-CATCGTGTTATCCTCTGTCAGC-3′	
	sifA	F:5′-ATGCCGATTACTATAGGCAATGG-3′	1,011
		R:5′-TTATAAAAAACAACATAACGCCG-3′	
	sseL	F:5′-GCCCCTTCCAGATTACTTTATATG-3′	269
		R:5′-TGCTTAATATATTTTCTTTGGTGG-3′	
	ssrA	F:5′-CTTACGATTACGCCATTTACGG-3′	706
		R:5′-ATTTGGTGGAGCTGGCGGGAGT-3′	
	ttrB	F:5′-AGCCTTCACAAATTGTCCATTG-3′	608
		R:5′-CCATCACCACCATCGGAATATG-3′	
SPI-3	misL	F:5′-AACACACTGTCACGGT-3′	458
		R: 5′-CAGACGAATCAACGAA-3′	
	rmbA	F:5′- CGCTGACGGTCGTTATCG-3′	454
		R: 5′-GTGGCGATGCGGCTATGG-3′	
	rhuM	F:5′-CATCGGCTGTACCCGACTAT-3′	222
		R: 5′-CAGCACGCTGATGAATGAGT-3′	
	sugR	F:5′- ATATCCGCTACCATCGCAAC-3′	152
		R:5′-TCAATGCTCAGACGGACTTC-3′	
SPI-4	siiD	F:5′-GAATAGAAGACAAAGCGATCATC-3′	655
		R: 5′-GCTTTGTTCACGCCTTTCATC-3′	
	siiE	F:5′-TTTTTTGCCGATCAAAATTCTGTA-3′	750
		R: 5′-TATACTATCATCTTTGCTACCGCT-3′	
	spi4H	F:5′-CGCTGACGGTCGTTATCG-3′	154
		R: 5′-TCAATGCTCAGACGGACTTC-3′	
SPI-5	pipC	F:5′-CGCCTCTTCTTCGGT-3′	145
		R: 5′-TATGCCATTGCCTGA-3′	

### Detection of Pathogenicity in Chickens

#### Lethal Test

Fifteen three-day-old SPF (Specific pathogen Free) chickens were randomly divided into five groups evenly. Four experimental groups were set up, and 1 negative control group was set up. Each chicken in the test group was intraperitoneally injected with 0.2 mL of the bacterial solution at a concentration of 4 × 10^8^ CFU/mL. The control group was intraperitoneally injected with the same amount of sterile PBS (phosphate buffer saline) solution. Death data were recorded after 7 consecutive days of observation.

#### Determination of Median Lethal Dose of Pathogenic Bacteria

Another 25 healthy 3-day-old SPF chicks were randomly divided into 5 groups of 5 in each group to avoid cross-infection. Four groups were taken as the experimental group. The concentration of bacterial suspensions was diluted into 4 × 10^9^ CFU/mL, 4 × 10^8^ CFU/mL, 4 × 10^7^ CFU/mL and 4 × 10^6^ CFU/mL, respectively, with sterile PBS.

The day of challenge was recorded as day 0. The onset time, symptoms and death time of the chickens were recorded twice a day. The dead chickens were dissected immediately, and the pathological changes were recorded. After 7 days of continuous observation, all chickens were euthanized. The bacteria were isolated and identified from dead chickens and the total number of deaths was recorded. The 50% lethal dose of bacteria (LD_50_) was calculated by the modified Kärber method.

$$\begin{array}{l} \text{lgL}{\text{D}}_{50}={\text{X}}_{\text{K}}-\text{d}\left(\sum \text{Pi}-0.5\right)\\ \text{Standard error S}\log\text{L}{\text{D}}_{50}=\text{d}\sqrt{\left(\text{ΣP}-\text{Σ}{\text{P}}^{2}\right)/\left(\text{n}-1\right)}\\ 95\text{\% confidence interval}\log^{-1}\left(\log\text{L}{\text{D}}_{50}\pm 1.96\times \text{S}\log\text{L}{\text{D}}_{50}\right) \end{array}$$

“*X*_*K*_” means the maximum logarithmic dose, “d” means the difference of logarithmic dose between two adjacent groups, “Pi” means the mortality rate, and “i” means the group number.

#### Histopathological Examination

The dead chickens were timely dissected. The pathological changes of tissues and organs were observed, and the hearts, lungs, livers, kidneys, spleens, bursa of fabricius and small intestine of the sick and dead chickens were collected, then fixed with 4% paraformaldehyde solution and stored at 4°C. Hematoxylin-eosin staining was performed on tissue samples. The histological changes were observed and analyzed under an optical microscope.

## Results

### Isolation and Identification of Bacteria

**Figure 1:**
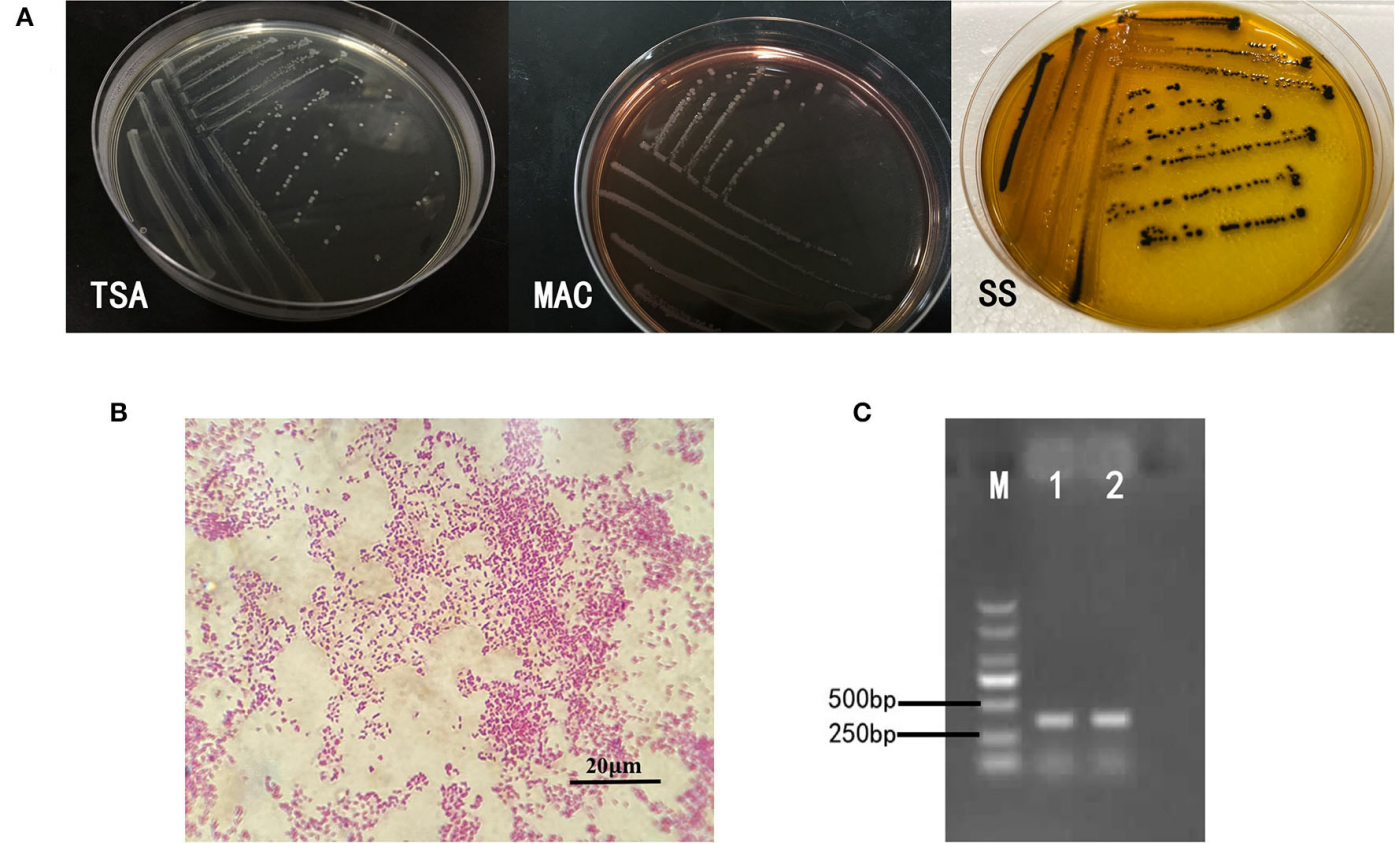
Isolation and identification of bacteria. **(A)** On the TSA medium, the bacteria presented a round, slightly raised, rounded and smooth surface, translucent colonies. Round translucent colonies with smooth edges appear on MAC identification medium. The strains on SS identification medium were round, slightly convex, with smooth and neat edges, and the center of the colony was black. **(B)** The bacteria were short shaped, without capsule and spores. Most of them are single. It was Gram-negative bacilli. **(C)** The invA gene-specific to *Salmonella* was used to amplify the DNA of the isolated strain by PCR, and the product size of the target fragment was 374 bp, which was the same as that of the reference strain of *S. enteritidis*.

Intestines of sick snakes were aseptically sampled and streaked onto TSA solid medium for pathogen culture and isolation. The suspected colonies were streaked on MAC solid medium and SS (Salmonella Shigella agar) solid medium for identification. The isolated strains showed different morphology on different mediums as shown in Figure 1A. After Gram staining, the isolated bacteria could be seen under ordinary optical microscope (Figure 1B). The invA gene-specific to *Salmonella* was used to amplify the DNA of the isolated strain by PCR, and the product size of the target fragment was 374bp (Figure 1C). In summary, these characteristics mentioned above are consistent with *Salmonella*.

### Identification of Serotypes

The serotype of *Salmonella* identified by the method of glass slide agglutination test was *S. enteritidis* (-, 9, 12: g, m: 7).

### Antimicrobial Susceptibility Test

The results of antimicrobial susceptibility tests are shown in Table 4. The results showed that the bacteria were resistant to most drugs, but sensitive to tetracycline and amikacin. The drug susceptibility results of ceftriaxone, kanamycin and neomycin were determined as intermediate.

**Table 4:** Antibiotic susceptibility of isolated bacteria.

**Susceptibility** **paper**	**Judgment standard**			**Diameter of bacteriostatic zone/mm**	**Results**
	**R**	**I**	**S**		
Florfenicol	≤ 12	13–18	≥19	0	R
Clindamycin	≤ 14	15–20	≥21	0	R
Polymyxin B	≤ 8	9–12	≥13	0	R
Cephalothin	≤ 14	15–17	≥18	0	R
Ofloxacin	≤ 12	13–15	≥16	0	R
Streptomycin	≤ 11	12–14	≥15	0	R
Gentamicin	≤ 12	12–14	≥15	0	R
Doxycycline	≤ 12	13–16	≥17	0	R
Cephradine	≤ 17	18–20	≥21	0	R
Tetracycline	≤ 11	12–14	≥15	18.2	S
Amikacin	≤ 14	15–16	≥17	26.0	S
Klaricid	≤ 9	10–14	≥15	0	R
Erythromycin	≤ 13	14–23	≥24	0	R
Norfloxacin	≤ 12	13–16	≥17	0	R
Amoxicillin	≤ 19	19–20	≥21	0	R
Kanamycin	≤ 14	15–17	≥18	17.0	I
Neomycin	≤ 12	12–17	≥18	13.8	I
Ciprofloxacin	≤ 15	15–21	≥20	0	R
Ceftriaxone sodium	≤ 13	14–21	≥22	13.2	I

*According to NCCLS drug susceptibility test standard, S, susceptible; R, resistant; I, intermediate*.

### Detection of Antimicrobial Resistance Genes

**Figure 2:**
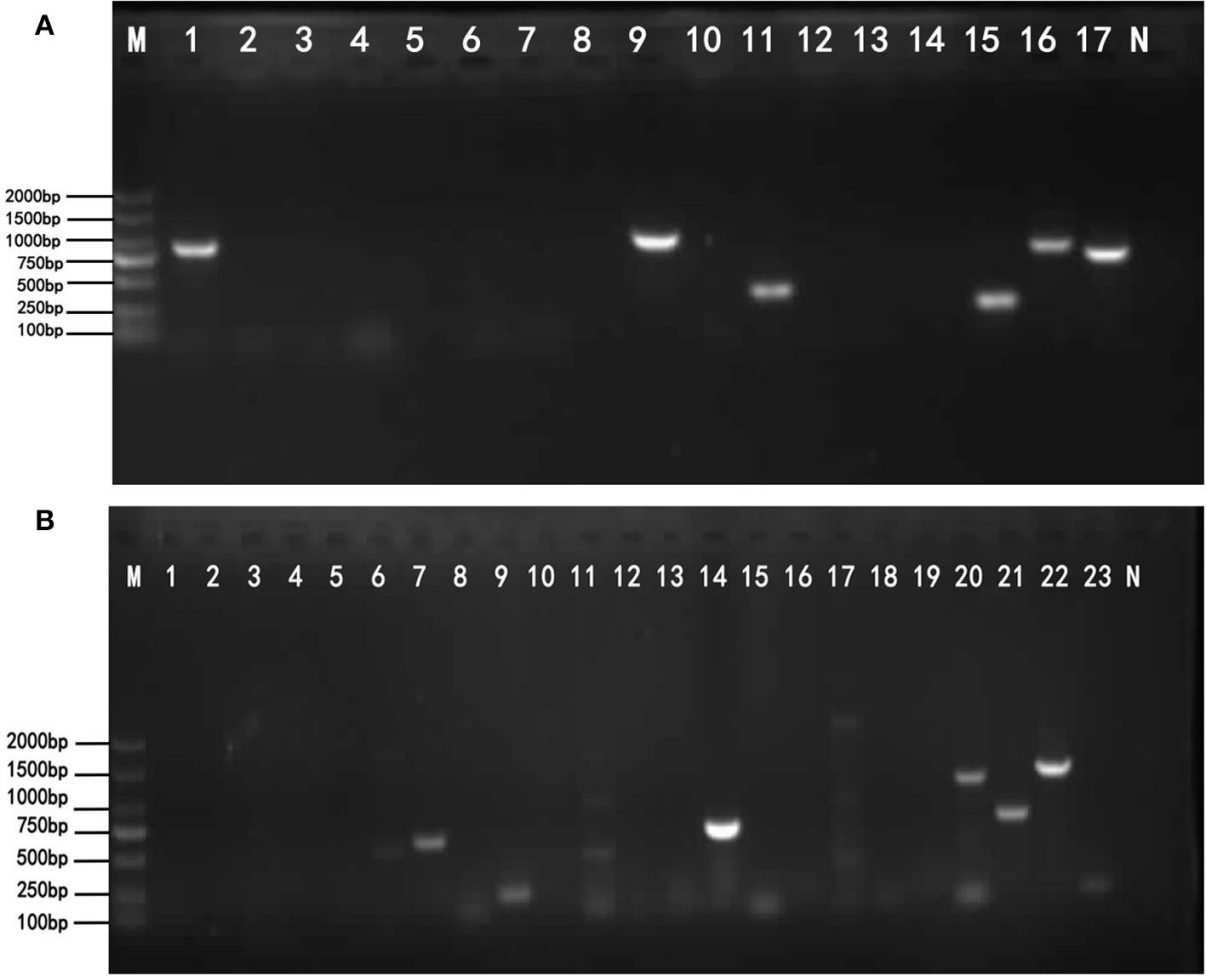
The results of drug-resistant genes and virulence genes. **(A)** The target resistant genes were amplified by PCR using the primers listed in Table 2. Lane M: DNA Marker DL2000 (from top to bottom is 2,000–1,500–1,000–750–500–250–100 bp). Lane 1–17: Resistance gene; Lane N: Negative control. The lane 1, lane 9, lane 11, lane 15, lane 16, and lane 17 show bright strips, which are, respectively, 643, 984, 343, 258, 822, and 722 bp. The results of the detected genes are shown in Table 5. **(B)** The target virulence genes were amplified by PCR using the primers listed in Table 3. Lane M: DNA Marker DL2000 (from top to bottom is 2,000–1,500–1,000–750–500–250–100 bp). Lane 1–23: virulence genes; Lane N: Negative control. The lane 7, 8, 9, 14, 20, 21, 22, and 23 show bright strips. The virulence genes corresponding to these positive bands are shown in Table 6.

DNA of the strains was extracted for PCR amplification of 17 drug-resistant genes. The amplification results of drug-resistant genes are shown in Figure 2A. Testing results show in Table 5. BlaTEM of β-lactams was found in the strains. The qnrD gene in 4-quinolones was found in the strains. The aadA_1_ gene in aminoglycosides was found in the strains. The catA_1_ in chloramphenicols was found in the strains. The genes sul I and sul II in sulfonamides were found in the strains. Sequencing results showed that the similarity between the six amplified drug-resistant gene sequences and the reference sequences in GenBank was more than 95% and they shared sequence homology.

**Table 5:** The positive detection of drug-resistant genes.

**Number**	**Resistant genes**	**Result**	**Number**	**Resistant genes**	**Result**
1	blaTEM	+	10	qnrS	−
2	blaSHV	−	11	aadA_1_	+
3	blaPSE	−	12	aad*A*_2_	−
4	blaCTM-M	−	13	tetA	−
5	blaOXA	−	14	tetB	−
6	qnrA	−	15	catA_1_	+
7	qnrB	−	16	sul I	+
8	qnrC	−	17	sul II	+
9	qnrD	+			

“*+” Indicates the gene was checked out; “−” means negative*.

### Detection of Virulence Genes

The 23 pairs of virulence genes were amplified by PCR from isolated *S. enteritidis*. The amplification results of virulence genes are shown in Figure 2B. The Table 6 shows the virulence gene testing results. Virulence genes avrA, iacP, prgK, ssrA, siiD (spi4D), siiE, spi4H, and pipC were found in the strains. The sequences of these 8 virulence genes were highly homologous with the virulence genes logged on GenBank, and their similarity was more than 95.5%. Therefore, the PCR amplification products of the above 8 virulence genes belonged to the virulence genes of *Salmonella*.

**Table 6:** The positive detection of virulence genes.

**Number**	**Resistant genes**	**Result**	**Number**	**Resistant genes**	**Result**
1	spvA	−	13	sseL	−
2	spvD	−	14	ssrA	+
3	invH	−	15	ttrB	−
4	sipA	−	16	misL	−
5	sopA	−	17	rmbA	−
6	sopD	−	18	rhuM	−
7	avrA	+	19	sugR	−
8	iacP	+	20	siiD (spi4D)	+
9	prgK	+	21	siiE	+
10	ssaB	−	22	spi4H	+
11	ssaQ	−	23	pipC	+
12	sifA	−			

“*+” Indicates the gene was checked out; “−” means negative*.

### Detection of Pathogenicity in Chickens

#### Lethal Results of the Strain

Twelve chickens were intraperitoneally injected with 0.2mL of fresh bacterial solution at concentration of 4 × 10^8^ CFU/mL. Within 4 h after challenge, some chickens died. Other chickens showed listlessness, loose fur, and body tremors after 48 h to challenge. The dead chickens had stiffened legs. Data were collected for 7 days of continuous observation. In the experimental group, 9 chickens died. While the control group had no obvious symptoms. So, the results showed that *S. enteritidis* had a 75% fatality rate in chickens.

#### Determination of LD_50_

The concentration of *S. enteritidis* administrated to chickens of each experimental group was 4 × 10^9^ CFU/mL, 4 × 10^8^ CFU/mL, 4 × 10^7^ CFU/mL, 4 × 10^6^ CFU/mL, respectively, corresponding to A1, A2, A3, and A4 groups. According to the data in Table 7, the modified Kärber method was used to calculate the 50% lethal dose of the strain.

**Table 7:** Experimental data on pathogenicity in chickens.

**Group**	**Dose**		**Number (*n*)**	**Death** **number**	**Mortality (*P*)**	**P^2^**
	**CFU**	**Log**				
A1	4 × 10^9^	9.6	5	5	1	1
A2	4 × 10^8^	8.6	5	5	1	1
A3	4 × 10^7^	7.6	5	2	0.4	0.16
A4	4 × 10^6^	6.6	5	0	0	0
					∑*P* = 2.4	∑*P*^2^= 2.16

The LD_50_ result was 10^7.7^ CFU/mL. While, standard error SlogLD_50_ was 0.2449. Ninety-five percent and Confidence Interval 95% CI.

#### Clinical Observation

After the strain was cultured for 10 h, the bacterial quantity of the strain was examined, which was 4 × 10^9^ CFU/mL by plate count method.

**Figure 3:**
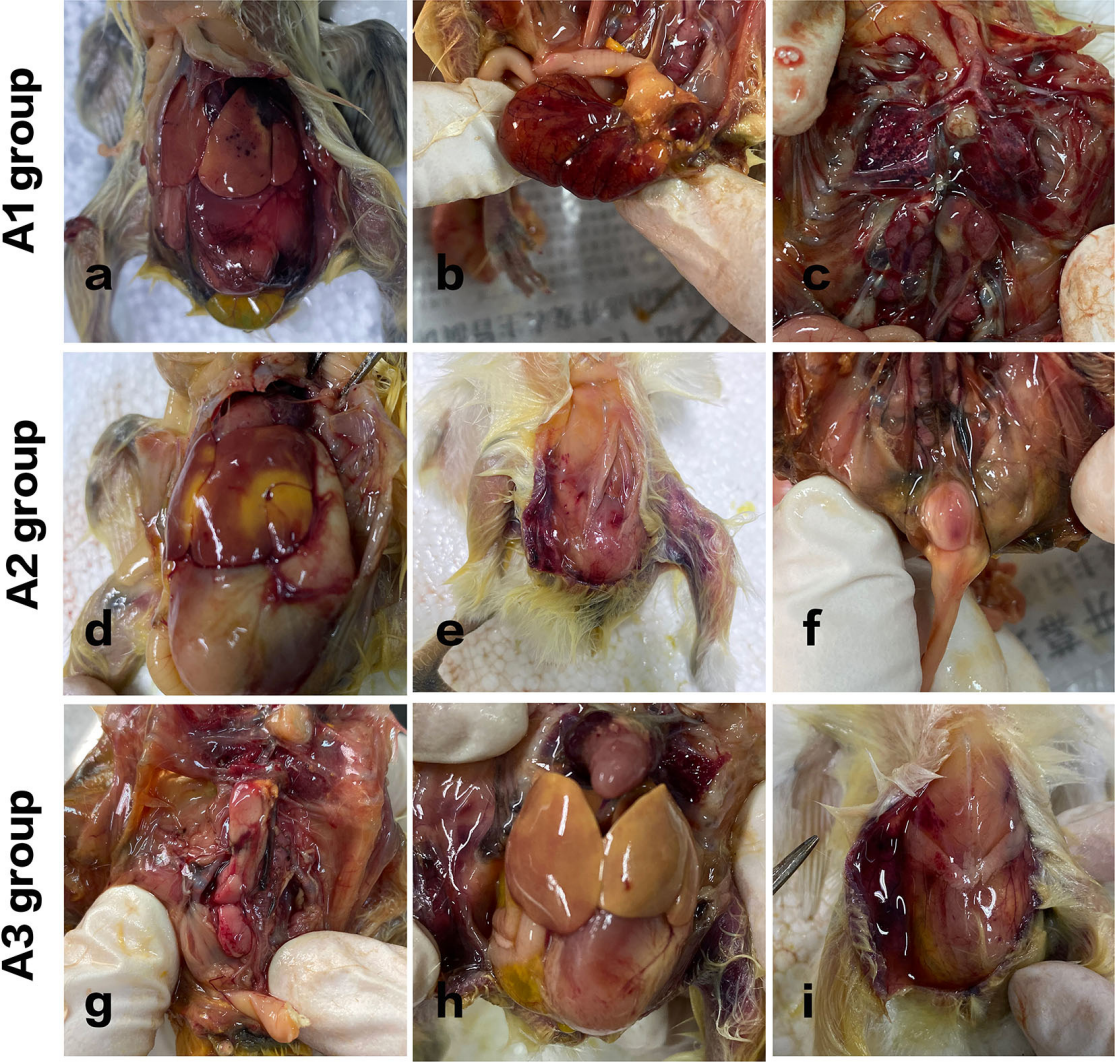
Pathological variation of chicks in different groups. A1 group: Acute septicemia, extensive abdominal bleeding, and redness. **(a)** Punctate bleeding points on the liver surface were seen. **(b)** The yolk sac was red and swollen. **(c)** Hyperemia in flamed lung tissues were seen. A2 group: **(d)** large yellow plaques and subcutaneous bleeding in the livers of the chickens. **(e)** Subcutaneous hemorrhage. **(f)** Enlarged bursa of Fabricius with hemorrhagic spots was observed. A3 group: **(g)** kidneys were swollen with spotty bleeding. **(h)** The liver turned yellow. **(i)** The peritoneum was in a viscous and gelatinous state.

After intraperitoneal administration of *S. enteritidis* suspension with different concentrations into chickens, clinical signs showed as followed (Figure 3).

In group A1, all the chickens died within 8 h after injection, and the course of the disease was urgent. Acute septicemia, extensive abdominal bleeding and redness, punctate bleeding points on the liver surface were seen. Kidneys were inflamed, and hyperemia in flamed lung tissues were seen. The control group of chickens showed no obvious redness and swelling in abdominal cavity.

In group A2, two chickens died 8 h after challenge, and the other three curled up in the corner, indolent, slow responsive, and anorexia. Another two died after 12 h administration. The rest of the chickens died within 24 h. And it turns out that large yellow plaques and subcutaneous bleeding in the livers of the chickens. Cecum was enlarged. Lungs were inflamed and congested. Enlarged bursa of Fabricius with hemorrhagic spots was also observed.

In group A3, one chicken died 2 days later after challenge, one chicken died on day 3. Figure 3 shows that the kidneys were swollen with spotty bleeding and the liver turned yellow. The intestinal tissue structure presented unclear boundaries and the peritoneum was in a viscous and gelatinous state.

In group A4, the chickens were bright, alert and responsive. They were euthanized on day 7. Compared with the A5 control group, the body weights were slightly lower.

#### Histopathological Examination

**Figure 4:**
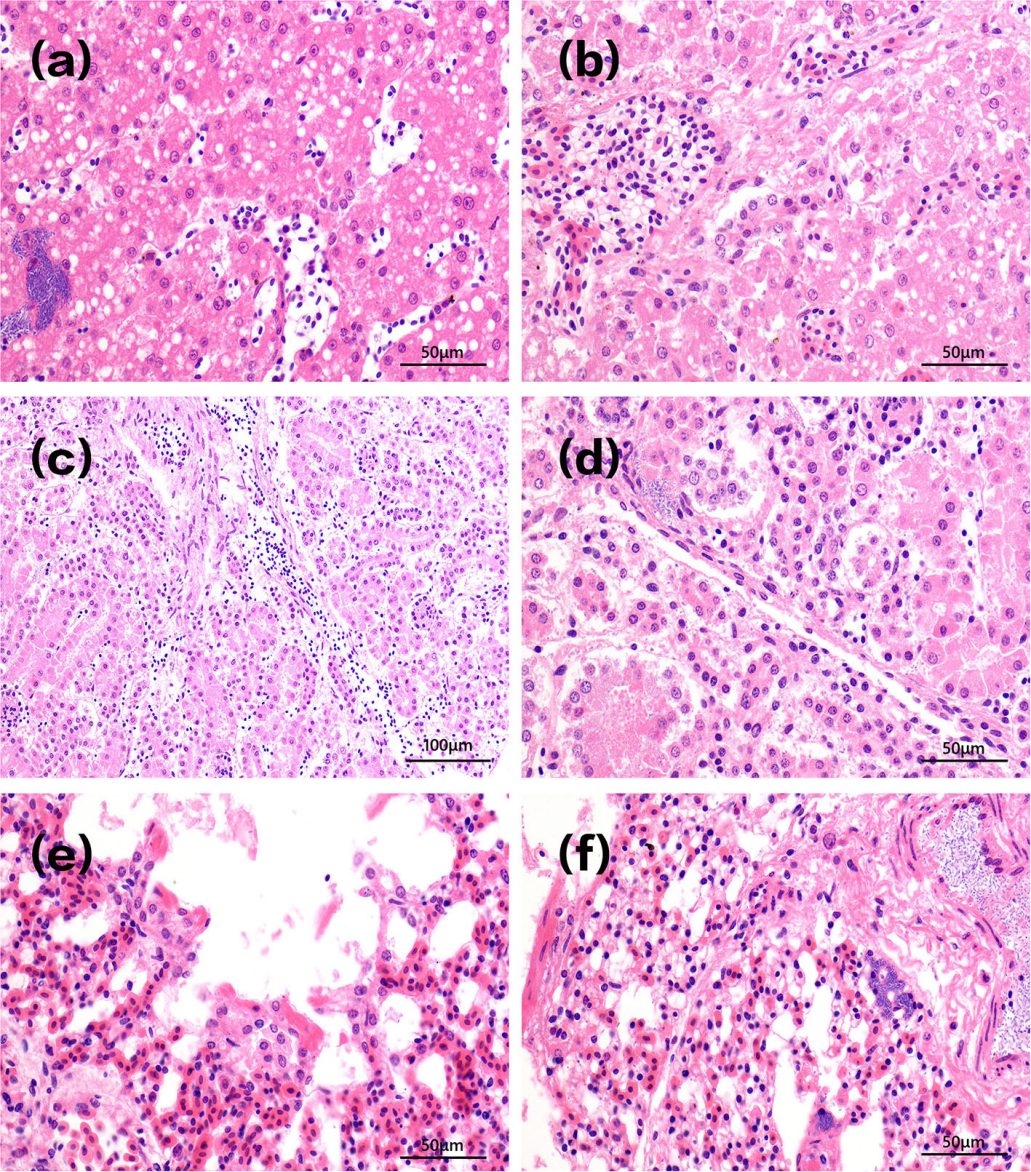
Pathological changes of tissues in infected dead chicks. Pathological changes of chick liver in acute A1 group are shown in **(a,b)**. The vacuoles in **(a)** were small in size, indicating that steatosis of liver cells was less severe. There is also a field of view of vacuolar degeneration of hepatocytes showed in **(b)**, in which the cellular matrix loosened and became an unstructured cystic space. Pathological changes of kidney in infected dead chicks were shown in **(c,d)**. **(c)** Inflammatory cells infiltrate chicken kidneys. **(d)** The epithelial cells of renal tubules were swollen and their structures were blurred, bacterial accumulation. Pathological changes of the lung at different stages of infection were shown in **(e,f)**. **(e)** Pulmonary capillary hyperemia, bacterial accumulation. **(f)** Alveolar serous fluid oozes out and alveolar epithelial cells proliferate and vacuoles appear in the cytoplasm.

Acute clinical symptoms developed in the chickens of group A1. Pathological slides of the livers showed that the hepatocytes were swollen with infiltrated inflammatory cells in the hepatic sinus. Fat droplets with varied sizes appeared in the cytoplasm of liver cells. The vacuoles in Figure 4a were small in size, indicating that steatosis of liver cells was less severe. There is also a field of view of vacuolar degeneration of hepatocytes showed in Figure 4b, in which the cellular matrix loosened and became an unstructured cystic space. The pathological changes of kidney in infected dead chicks showed numerous inflammatory cells oozing out in acute dead chickens' kidneys (Figure 4c), and the epithelial cells of the renal tubules were blurred, with bacterial accumulation in Figure 4d.

Figure 4e shows infected lungs that congested pulmonary septum capillaries, a large number of neutrophils exudate, and rod-shaped bacteria in alveolar space can be seen in infected chickens that died at day 1. A large number of serous exudates from the alveolar cavity, mixed with more neutrophils, red blood cells, and exfoliated epithelial cells was seen 3 days after infection. After 10 days of injection, the alveolar structures were almost indistinguishable, alveolar epithelial cells proliferated, and alveolar cells showed vacuoles of different sizes in Figure 4f.

## Discussion

*Salmonella enterica* is considered an important zoonotic enterogenic pathogen. *S. enterica* can be divided into 6 subspecies and more than 2,600 serotypes, which can cause different degrees of infection (17). According to previous reports in other countries, frequent *Salmonella* infection in reptiles has been confirmed, suggesting that reptiles are also natural hosts for *Salmonella* (25–27). Reptiles are known to transmit *Salmonella* intermittently (28) and shed into the environment irregularly. In addition, bacteria-carrying fecal sprinkled on the surface of the earth will flow along with the rain and contaminate the soil, land crops, or residential water (29–31), which is difficult to eradicate through antibiotic treatment. Therefore, there may be risks for both carriers and people in close contact with these animals (32).

Contaminated water or food is thought to be the main source of *Salmonella* transmission. Currently, *Salmonella* prevention mainly focuses on water and food safety. Developed countries and areas such as Europe and the United States are able to implement procedures to prevent *S. enteritidis* transmission while developing countries and regions still need improvements. In addition to becoming *Salmonella*-contaminated food, edible snakes can also pollute water sources and transmit the bacteria to other animal farms. Infected chickens can also be the source for Salmonella infection in humans, leading to global public health concern (33).

Current published studies mostly focused on the increasing popularity of reptiles as pets and their possible role as hosts for pathogenic microorganisms. Frequent touch between reptiles and humans is considered a public health problem because these animals have been identified as carriers of zoonotic infectious diseases, especially *Salmonella* associated with human salmonellosis (32). In a Brazilian study, *Salmonella* isolated from reptiles was identified with a karyotype that has been reported to infect humans (34). Little is known about how reptiles carry pathogenic microorganisms (35). The information about major subspecies and serotypes of *Salmonella* isolated from reptiles is limited. *Salmonella* is isolated from snakes often more than other reptiles (36), possibly because of the snakes' dietary habits. *Salmonella* spreads fastest among carnivorous reptiles, such as snakes (37). This study investigated the incidence of pneumonia on a chicken-fed Chinese snake farm. It is likely that *Salmonella* from chicken meat accumulates in the snakes. Chicken was originally thought to be the main host of *Salmonella* (11). Chicken meats the potential source to infect this snake farm. Therefore, the pathogenicity of *S. enteritidis* isolated from snake pneumonia was studied in chickens.

In order to explore the pathogenicity of *S. enteritidis* isolated from snake pneumonia, the strain was administrated intraperitoneally in 3-day-old chicks, and half of the lethal dose of the strain was examined, as well as the pathological changes of each organ of the chicks. In the bacterial strain infection model, acute sepsis was observed in the highest dose group A1, which was consistent with previous research results (38–41). The natural infection of *Salmonella enterica* leads to systemic and persistent infection, which potentially causes bacteria to spread to other hosts, including in the poultry industry (42–44). Obvious redness and congestion were also seen in the lungs. Due to the rapid onset and short course of the disease, no respiratory symptoms were observed in the chickens. Bacteria solution was administrated intraperitoneally, and half of the lethal dose was 10^7.7^ CFU/mL. For the high-dose A1 and A2 groups, the mortality rate was 100%, with all deaths occurring within 24 h. In addition, the lower dose groups A3 and A4 showed decreased weight gain after *Salmonella* infection, and the body weight began to decrease 5 days after infection. Chickens challenged with *Salmonella* showed symptoms of anorexia, diarrhea with high mortality. Chickens that had made through the infection also suffered stunted growth. Results of the pathogenicity test showed that this strain is highly pathogenic in warm-blooded animals with a low median lethal dose, and the potential human transmission route needs further study. Existing studies have shown that *Salmonella* from reptile sources is more likely to transmit to human beings than *Salmonella* from non-reptile sources. Therefore, reptile-related *Salmonella* should be considered to be a global threat to public health (12) and worthy of vigilance.

PCR detection of *Salmonella* virulence genes has been widely used as a predictive measurement of *Salmonella* virulence. It is known that there are significant genetic variations in virulence gene sequences between *Salmonella* serotypes. This variability in the genome sequence may translate into changes in function, leading to differences in pathogenicity. However, it must be noted that having one or more virulence genes does not confer pathogenicity on a strain unless the strain acquires the appropriate virulence gene combination to cause disease in a particular host species (45). The *Salmonella* type III secretory protein system, composed of proteins encoded by the virulence island gene, also plays a key role in the pathogenicity of *Salmonella* (46). The low dose of LD_50_ and the presence of multiple virulence genes indicate that the number of virulence genes in the strain is related to the pathogenicity.

Antimicrobial susceptibility test showed that the isolated *Salmonella enteritidis* was resistant to the vast majority of the 19 selected antimicrobials, and some do not appear bacteriostatic ring at all. It is only sensitive to tetracycline, amikacin, kanamycin, neomycin, and ceftriaxone. Currently, 4-quinolonesand β-lactams are mainly used to treat *Salmonella* infection. Notably, cephalosporin is an antimicrobial agent that can be used to treat human urinary tract infections (47, 48), and ciprofloxacin is a quinolone that is of great importance in human medicine and is mainly used to treat severe infections (49). However, in this study, isolated *Salmonella* were resistant to the selected third generation quinolones. The isolated strains also showed low sensitivity to β-lactams antibiotics. Potential drug resistance could occur in human beings with salmonellosis if such strains transmitted into people (50).

The antimicrobial resistance of bacteria has a certain relationship with the resistance genes carried by bacteria. The test results showed that the resistance phenotype of the strains was basically in consistent with the detection rate of antimicrobial resistance genes. For each selected drug-resistant gene category, the corresponding drug-resistant genes were detected in β-lactams, 4-quinolones, Aminoglycosides, Tetracyclines, Chloramphenicols, and Sulfonamides. As time goes on, bacterial resistance rates will also continue to rise, and antimicrobial resistance profiles will become more complex, perhaps leading to more intense foodborne salmonellosis. The chromosomal resistance can be transmitted to the next generation and also at the level of different genera, which greatly expands the number and range of drug-resistant bacteria, leading to the increasingly serious phenomenon of *Salmonella* resistance. At the same time, the resistance of pathogenic *Salmonella* strains of the same animal source isolated from different countries or regions in the same time period or in the same country or region in different time periods also varies, that is, the expression has certain locality and timeliness (20, 51). Pathogenic strains from different animal sources vary even more. Therefore, it is necessary to strengthen the study of antimicrobial resistance phenotype of *Salmonella*. The recombination of virulence genes and antimicrobial resistance gives Enterobacteriaceae a survival advantage when exposed to a drug environment that is unfavorable to growth, posing a serious threat to public health. This raises the potential risk to human health from these bacteria. In summary, this is the first report on *Salmonella enteritis* strains isolated from edible snakes with pneumonia in China. The isolated strains were extensively antimicrobial resistant, and potentially pathogenic. Thereby, rising public health concerns. Further studies should focus the underlying mechanisms involved in the possible spread of these strains to humans.

## Data Availability Statement

All datasets generated for this study are included in the article/supplementary material.

## Ethics Statement

This animal study was reviewed and approved by Institution's Animal Care and Health and Supervisory Committee of Huazhong Agriculture University, Wuhan, China.

## Author Contributions

YS and YX: conceived and designed the experiments, analyzed the data, and wrote the paper. YS: provided the material and revised the article. YX and HL: performed the experiments. All authors contributed to the article and approved the submitted version.

## Conflict of Interest

The authors declare that the research was conducted in the absence of any commercial or financial relationships that could be construed as a potential conflict of interest.

## Abbreviations

CFU: Colony-Forming Units
CLSI: Clinical and Laboratory Standards Institute
K-B: Kirby-Bauer
LD_50_: Median Lethal Dose
PBS: Phosphate Buffered Saline
*S. enterica*: *Salmonella enterica* ssp. Enterica
S. enteritidis: Salmonella enterica serovar Enteritidis
SPF: Specific Pathogen Free
SS: Salmonella Shigella Agar
*S. typhimurium*: *Salmonella Typhimurium*
TSA: Tryptose Soya Agar
TSB: Tryptic Soy Broth.
